# Effects of activities participation on frailty of older adults in China

**DOI:** 10.3389/fpubh.2024.1483166

**Published:** 2024-11-20

**Authors:** Zihan Ni, Xiuyuan Zhu, Yuxin Shen, Xiaoying Zhu, Shiyu Xie, Xiaoguang Yang

**Affiliations:** ^1^School of Elderly Care Services and Management, Nanjing University of Chinese Medicine, Nanjing, China; ^2^Nossal Institute for Global Health, School of Population and Global Health, The University of Melbourne, Melbourne, VIC, Australia; ^3^Chinese Hospital Development Institute, Shanghai Jiaotong University School of Medicine, Shanghai, China

**Keywords:** frailty, physical activity, social activity, economic activity, information activity, sleep activity, older adults

## Abstract

**Objective:**

Frailty represents a significant health challenge among older adults, necessitating effective interventions to enhance their overall wellbeing. This study aims to investigate the impact of various types of activity participation on frailty in older adults and to elucidate their intrinsic associations, thereby providing a basis for targeted interventions.

**Methods:**

This study constructed a classification of activities based on the framework proposed by the WHO regarding functional ability in healthy aging, innovatively dividing activities into five categories: physical activity, social activity, economic activity, information activity and sleep activity. Utilizing data from the China Health and Retirement Longitudinal Study (CHARLS 2020), the research employed multiple linear regression and mediation analysis to explore the effects of these activities on the frailty status of older adults and their underlying mechanisms. Furthermore, propensity score matching was conducted to robustly test the regression results.

**Results:**

The study found that physical activity (β = −0.006, *p* < 0.01), social activity (β = −0.007, *p* < 0.01), economic activity (β = −0.017, *p* < 0.01), information activity (β = −0.040, *p* < 0.01) and sleep activity (β = −0.044, *p* < 0.01) all had significant positive effects on the frailty status of older adults. Additionally, sleep activity mediated the relationship between physical activity and frailty status, accounting for 4.819%. Social activity mediated the relationship between information activity and frailty status, accounting for 7.692%.

**Conclusion:**

Older adults should enhance their participation in various activities to alleviate frailty. This can be further improved through the following three aspects: engaging in moderate physical exercise, fostering and promoting awareness of volunteer services, and popularizing the use of information technology.

## 1 Introduction

With the intensification of the aging population process, frailty among the older adults is becoming an increasingly prominent health challenge. Frailty is a common and complex geriatric condition, often defined as a decrease in physiological reserve and reduced resistance to stressors caused by cumulative declines in multiple physiological systems (1–3). Frailty is considered to be a dynamic process that worsens with age (4), leading to adverse consequences for the physical and mental health of older adults. During the period of frailty, the probability of older adults developing other diseases is 12%−24%, with a incidence rate as high as 46% to 49% in the early stage of frailty (5). Besides, frailty is closely associated with adverse health outcomes such as cognitive impairment (6), disabilities (7), hospitalization (8), and death (9). Fortunately, frailty is a reversible process, and early identification of risk factors as well as proactive interventions can delay the progression of older adults from pre-frailty to frailty (10). Among these factors, participation in activities stands out as a particularly significant influence on frailty in older adults (11–13). Activity theory posits that older adults with higher levels of activity tend to exhibit greater life satisfaction (14) and stronger social adaptation compared to those with lower activity levels. This positive effect can be attributed to the supportive roles provided by engagement in activities (14), which help older adults maintain a positive self-concept, prevent brain degeneration, reduce dysfunction, enhance positive emotions, and improve quality of life. Additionally, these activities bolster the ability to cope with environmental changes and organic frailty (14–16). Existing research indicates that, even after controlling for demographic variables, engagement in activities significantly elevates the overall health status of older individuals and mitigate the onset of frailty (17–19). However, the impact of different types of activities on frailty in older adults may vary (18). Therefore, it is essential to further delineate the types of activities and investigate the specific effects and underlying mechanisms of these activities on frailty in older adults.

**Figure 1:**
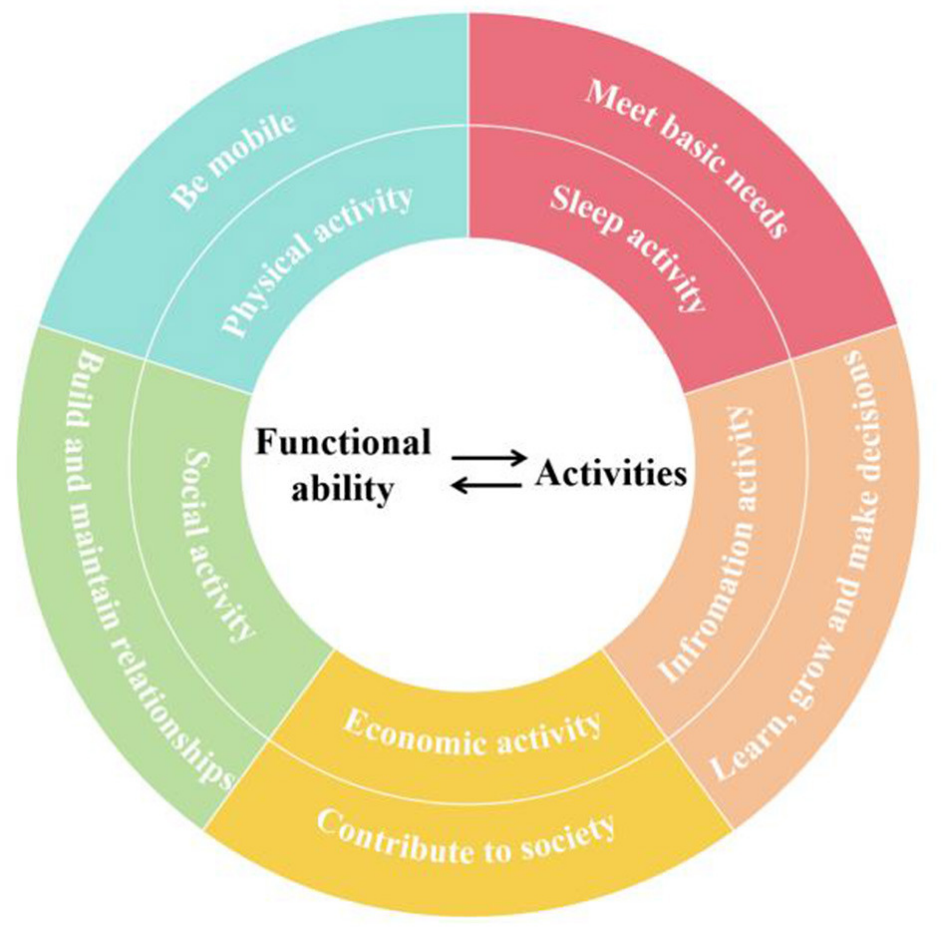
Framework of activities of older adults.

The World Health Organization (WHO) states that healthy aging is the process of developing and maintaining the functional ability that enables wellbeing in older individuals. Functional ability encompasses a person's capacity to move independently, build and maintain relationships, contribute to society, make decisions, learn and grow, as well as meet their basic needs (20). Older individuals demonstrate their functional ability through activity participation, which in turn affects their health status (21). Based on the WHO's framework for functional ability in older individuals, we categorize their activities into five types: physical activities, social activities, economic activities, information activities and sleep activities, as illustrated in Figure 1.

Physical activity refers to activities that require energy expenditure, muscle movement, and physical labor. It is commonly classified into three categories: vigorous physical activities (VPA), moderate physical activities (MPA), and low-intensity physical activities (LPA) (22). Physical activity can mitigate and delay several adverse health outcomes associated with frailty in older individuals, such as cognitive impairment, muscle loss, and depression (23). Consequently, it is considered one of the most effective methods for improving the quality of life and functional abilities in older individuals (24), and it may even serve as a predictor of frailty (25). Higher levels of moderate-to-vigorous physical activity (MVPA) are effective in improving frailty (26), reducing its incidence (27), preventing mobility disorders in frail older individuals (28), and counteracting the negative effects of sedentary behavior (29). However, despite the positive impacts of physical activity on the health of older individuals, it also increases the risk of falls and fractures due to decreased bone mineral density associated with aging (30). Therefore, Kwok et al. encouraged retirees to engage in physical activities following health activity guidelines to minimize these risks (31).

Social activity refers to various communication and interaction behaviors in social life that older adults participate in, involving visiting or socializing with friends, playing mahjong and chess, etc. After retirement, older adults are freed from the constraints of work schedules and social activities, providing them more leisure time. In this new stage of life, they can meet their needs for fulfillment through active social engagement, facilitating a smooth transition from work to retirement (10). Social activities are capable of promoting older adults to establish social networks, receive emotional support and recognition, enhance self-efficacy and enhance stress resistance, thereby reducing the risk of illness as well as improving quality of life (32). Conversely, insufficient engagement in social activities may lead to feelings of loneliness and social isolation among older adults, increasing their risk of frailty. Existing researches have indicated that living in socially active neighborhood is related to lower levels of frailty, highlighting the crucial role of enhancing social activity participation in frailty prevention (33). Most researchers suggested that social activities may impact the frailty status of older adults and researches primarily focused on impacts of different categories of activities (34). Some existing researches indicate that engaging in group games, joining sports clubs and participating in voluntary work can effectively reduce the risk of frailty among older adults. However, some basic social interactions, such as interacting with friends and joining community organizations, have not been proved to have a positive impact on alleviating frailty in older adults (35). Takatori et al. suggested that social factors have no significant improvement effect on frailty in older adults, but social activities based on the promotion of exercise could improve the status of older adults before frailty (36).

Economic activity means various behaviors undertaken by older adults for surviving and developing. Engaging in economic activities may lead to two distinctly different living conditions for older individuals, thereby affecting their physiological and psychological status in different ways. Academic researches on work and frailty in older adults has yielded mixed findings. Research by Jung et al. in the field of productive activities (including volunteer service, paid work, and childcare) showed that after controlling for age, disability, and cognition, work was irrelevant with the likelihood of accumulating frailty (37). However, another research showed that both shift work and low payment have negative effects on the frailty status of older adults. The disruption of circadian rhythms caused by shift work can exacerbate frailty in older adults (38). Retirement may have a protective effect on the health of the older adults who are engaged in low-paid work because of their negative social psychology, which in turn increases their frailty (39). A longitudinal study in the UK supported the aforementioned views, indicating that leaving paid employment before reaching retirement age is beneficial for slowing the progression of frailty in men, and continuing to work does not provide long-term health benefits (40). Furthermore, another study classified work frequency into light, moderate, and vigorous, analyzing their impacts on frail older adults. The results showed that moderate work significantly decreased the pre-frailty rate in older adults (41). What's more, work can also be categorized into intellectual and physical work, both of which have been shown to have positive effects on frailty in older adults (18).

Information activity means utilizing information and communication technologies by older adults. The choice of whether or not to use the internet can reflect the degree of social adaptation and attitude toward societal changes among older adults, which can further influence their living conditions and mindset. The rapid development of information and communication technology (ICT) provides opportunities for users to connect with society, and has become an important mean to help older adults improve frailty status. The use of ICT by older adults can facilitate connections with family members, potentially improving their psychological wellbeing. The lifting effect may be more pronounced in frail older adults (42). Meanwhile, A cross-sectional study indicated a negative correlation between frailty and the use of ICT, with non-use of ICT being a predictor of frailty, and concluded that older women who did not use ICT were more likely to be frail (43).

Sleep activity pertains to the sleeping behavior of older adults. Research on the impact of sleep activity on frailty in older adults can be divided into two categories: sleep quality and sleep duration. Older adults with poor sleep quality are at a higher risk of physical frailty (44). Specifically, sleep-related insomnia can lead to decreased physical performance in older adults (45), resulting in a 66% higher likelihood of frailty compared to peers (46). Additionally, after controlling for covariates such as demographic information, lifestyle, and health-related conditions, poor sleep quality is highly associated with the probability of cognitive frailty (47). Existing studies of sleep duration on frailty in older adults have shown a non-significant association between short sleep duration and risk of frailty (48), with only excessive sleep duration being significantly associated with a high risk of cognitive frailty (49). This may be attributable to the U-shaped relationship observed between sleep duration and all-cause mortality, wherein the lowest risk is associated with a sleep duration of 5.8 h from sleep onset (50).

Different types of activities may interact with each other, thereby influencing frailty in older adults. Regular physical activity promotes relaxation and energy consumption (51), and has been proposed as an effective non-pharmacological treatment option to improve sleep in older adults (52). Existing studies have shown that physical activity is associated with sleep quality in older adults (53), with those engaging in higher levels of physical activity exhibiting better sleep quality (54) and regularity (55). Regular moderate-intensity physical activity in older adults significantly improves sleep outcomes such as sleep quality, sleep disorders, and sleep duration (56). Improved sleep conditions may further slow down the progression of frailty in older adults. Therefore, it is hypothesized that sleep activity may have a mediating effect between physical activity and frailty in older adults. The emergence of ICT has created new possibilities for older people to stay socially connected (57). The use of ICT by older adults can enhance communication efficiency and frequency with family members and friends, facilitating closer connections between them (58). Furthermore, ICT can help older adults overcome spatial limitations, enabling them to connect with strangers online and even develop offline relationships, thereby expanding their social circles (59). Given that participation in social activities, both online and offline may influence frailty in older adults, it is hypothesized that social activity may mediate the relationship between information activity and frailty in older individuals.

All in all, academic researches on the effects of different activity types on frailty in older adults remain inconclusive. There might be variations in the effects of different activity types on frailty among older adults, and may exist correlations between different types of activities that subsequently influence frailty. However, current academic researches on the impact of various activities on frailty primarily focuses on the effects of single or limited categories of activities (18, 60), which has resulted in a fragmented body of studies. There is a lack of comprehensive and systematic research examining the influence of the main activities that older adults frequently engage in during their daily life on frailty. Therefore, this study constructed a classification of activities based on the framework proposed by the WHO regarding functional ability in healthy aging, innovatively dividing activities into five categories: physical activity, social activity, economic activity, information activity and sleep activity. In addition, this study differs from the majority of previous research that concentrates on the mechanisms of alleviating frailty in older adults by improving physiological and psychological factors (61, 62). The findings will clarify the individual and interactive effects of various activities on frailty, deepening and expanding existing academic researches on this topic. Furthermore, based on the results of this research, the study will further provide practical recommendations for improving frailty in the daily lives of older adults, ultimately enhancing their overall wellbeing. Based on the analysis above, this study proposes the following hypotheses:

H1: Physical activity can effectively mitigate the frailty in older adults, but participation in physical activities of different intensities has varying impacts on frailty.
H2: Social activity can effectively mitigate the frailty in older adults and participation in different social activities also has varying effects on frailty conditions.
H3: Economic activity can effectively mitigate the frailty in older adults.
H4: Information activity can effectively mitigate the frailty in older adults.
H5: Sleep activity can effectively mitigate the frailty in older adults.
H6: Sleep activity mediates the impact of physical activity on frailty in older adults.
H7: Social activity mediates the impact of information activity on frailty in older adults.

## 2 Methods

### 2.1 Data and participants

The study utilized the data from the fifth phase (2020) of the China Health and Retirement Longitudinal Survey (CHARLS), which is an ongoing longitudinal cohort study. The aim of CHARLS is to collect a high-quality set of micro data representing Chinese households and individuals aged 45 and above, investigating 150 counties and 450 communities (villages) across 28 provinces (autonomous regions, and municipalities) in China. The CHARLS National Baseline Survey was launched in 2011 and was tracked 2 to 3 years. In order to guarantee the unbiased and representativeness of the survey, CHARLS set a filtering section that can exclude invalid samples. CHARLS also conducted the sample through four phases at the county (district)-village (residential)-household-individual level. CHARLS used the Probability Proportional to Size (PPS) method for sampling at two levels: county (districts)-village (residential). By the completion of the follow-up in 2020, the sample has covered a total of 19,000 respondents from 11,400 households, making it a good-quality dataset that includes individual basic information, family structure, health status, economic conditions, social security, and other aspects. All participants in the study signed informed consent forms, and the protocol has been approved by the Ethics Committee of Peking University.

**Figure 2:**
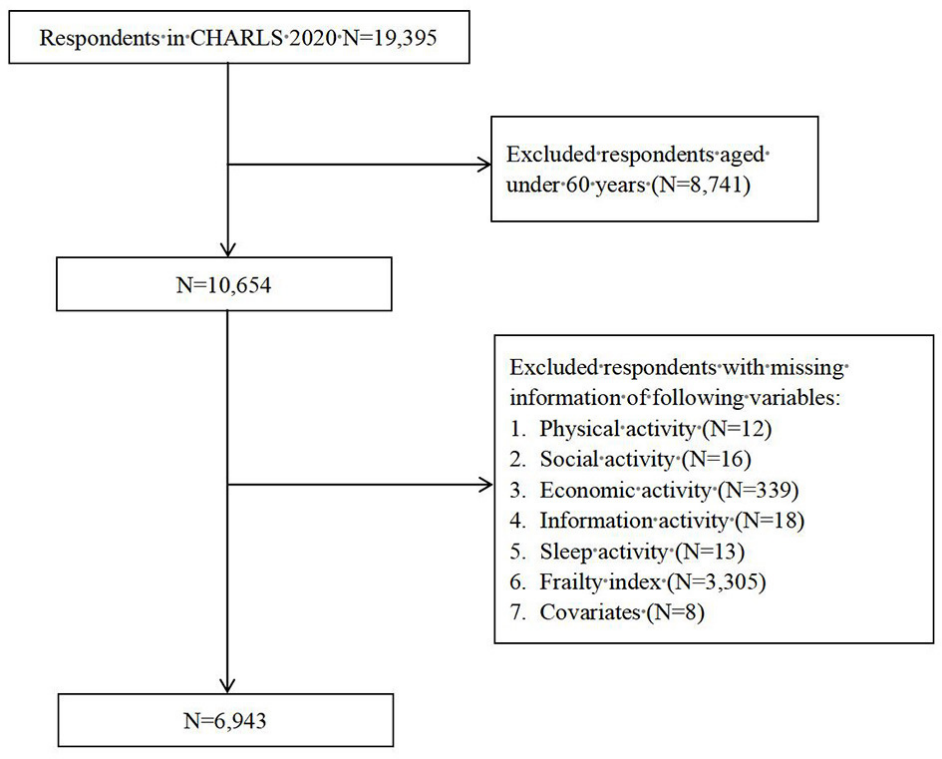
Respondents' flow in the study.

Since the research object was the older adults in this study, 8,741 respondents under the age of 60 were excluded. Among the remaining respondents, 12 were missing information on physical activity, 16 were missing information for social activity, 339 were missing information for economic activity, 18 were missing information for sleep activity, 3,305 were missing information on frailty index and 8 were missing information for covariates. Six thousand nine hundred and forty three respondents were finally included in our research and the sample selection process is depicted in Figure 2.

### 2.2 Dependent variable

The dependent variable in this study was frailty, which was measured by frailty index (FI). The index was consistent with previous studies (63, 64). It encompassed 8 domains, including chronic diseases, self-reported health, ADL, IADL, depression, and cognitive, comprising a total of 37 indicators, which were derived from self-report and objective measurement of the study participants. The specific items were shown in Table 1. FI was calculated by dividing the sum of scores by the total number of items, with scores ranging from 0 to 1, and a higher score indicated a higher level of frailty among older adults.

**Table 1:** Construction of frailty index indicator system.

**Domains**		**Description of the items**	**Cut-off value**
Chronic diseases	1	Self-reported physician diagnosed hypertension	Yes = 1, No = 0
	2	Self-reported physician diagnosed dyslipidemia	Yes = 1, No = 0
	3	Self-reported physician diagnosed diabetes	Yes = 1, No = 0
	4	Self-reported physician diagnosed cancer	Yes = 1, No = 0
	5	Self-reported physician diagnosed chronic lung diseases	Yes = 1, No = 0
	6	Self-reported physician diagnosed liver disease	Yes = 1, No = 0
	7	Self-reported physician diagnosed heart attack	Yes = 1, No = 0
	8	Self-reported physician diagnosed stroke	Yes = 1, No = 0
	9	Self-reported physician diagnosed kidney disease	Yes = 1, No = 0
	10	Self-reported physician diagnosed stomach or other digestive diseases	Yes = 1, No = 0
	11	Self-reported physician diagnosed any emotional, nervous, or psychiatric problems	Yes = 1, No = 0
	12	Self-reported physician diagnosed memory-related disease	Yes = 1, No = 0
	13	Self-reported physician diagnosed arthritis or rheumatism	Yes = 1, No = 0
	14	Self-reported physician diagnosed asthma	Yes = 1, No = 0
Self-reported health	15	Self-reported rating of health status	Poor/very poor=1, fair/good/very good=0
ADL	16	Difficulty with dressing	Yes = 1, No = 0
	17	Difficulty with bathing or showering	Yes = 1, No = 0
	18	Difficulty with eating	Yes = 1, No = 0
	19	Difficulty with getting into or out of bed	Yes = 1, No = 0
	20	Difficulty with using the toilet	Yes = 1, No = 0
	21	Difficulty with controlling urination and defecation	Yes = 1, No = 0
IADL	22	Difficulty with doing household chores	Yes = 1, No = 0
	23	Difficulty with preparing hot meals	Yes = 1, No = 0
	24	Difficulty with shopping for groceries	Yes = 1, No = 0
	25	Difficulty with taking medications	Yes = 1, No = 0
	26	Difficulty with managing money	Yes = 1, No = 0
Depression	27	Bothering by things much of time	Yes = 1, No = 0
	28	Having trouble keeping mind on things much of time	Yes = 1, No = 0
	29	Feeling depressed much of time	Yes = 1, No = 0
	30	Feeling that everything is an effort much of time	Yes = 1, No = 0
	31	Feeling hopeful about the future much of time	No = 1, Yes=0
	32	Feeling fearful much of time	Yes = 1, No = 0
	33	Sleeping is restless much of time	Yes = 1, No = 0
	34	Feeling happy much of time	No = 1, Yes=0
	35	Feeling lonely much of time	Yes = 1, No = 0
	36	Feeling that could not get going much of time	Yes = 1, No = 0
Cognition	37	Sum of orientation test score, memory test score and calculating test score/30	Continuous, ranging from 0 to 1

### 2.3 Independent variables

This study selected different types of activities as independent variables, including physical activity, social activity, economic activity, information activity, and sleep activity. For physical activity, the study evaluated its impact on frailty based on varying intensity levels. The research also explored the impact of different types of social activities on frailty. For economic and informational activities, the study measured their impact on frailty based on participation status. Regarding sleep activities, the research evaluated the impact on frailty based on the duration of sleep.

Referring to previous literature (22), physical activity among older adults was categorized into three levels based on intensity: low-intensity physical activity (LPA) such as walking, including walking for work or from one place to another at home, as well as walk for leisure, exercise, or entertainment. Moderate physical activity (MPA), such as carrying light objects, regular-speed cycling, mopping, brisk walking, etc., leading to faster breathing than usual. Vigorous physical activity (VPA) that cause rapid breathing, such as lifting heavy objects, farming, aerobics, fast cycling, etc. Participants were asked to report the intensity, frequency, and duration of their physical activity per week. The frequency ranged from 0 to 7 days per week, and the daily duration of physical activity was divided into five categories, i.e., no physical activity physical activity = 0 min, physical activity <30 min, 30–119 min, 120–239 min, and >240 min. The total of physical activity (TPA) was calculated by multiplying the daily duration of each type of physical activity by its weekly frequency. To further standardize the measurement of physical activity, the study utilized metabolic equivalent value (MET). Based on previous research, 1 MET represents resting oxygen consumption, while VPA corresponds to 8 MET, MPA to 4 MET, and LPA can be expressed as 3.3 MET. Total physical activity (TPA) was calculated by the sum of MET of VPA, MPA and LPA. For statistical convenience, the TPA was divided by 60 to calculate the MET hours per week and presented in the final data form using the LN (TPA+1).

Social activity among older adults included seven categories: (1) visiting or socializing with friends, (2) playing mahjong, chess, cards, or attending community activities, (3) providing unpaid help to non-cohabiting relatives, friends, or neighbors, (4) participating in sports, social, or other types of clubs, (5) participating in community-related organizations, (6) participating in volunteer or charity activities or caring for non-cohabiting patients or disabled persons, (7) attending school or training courses. The study measured the dimension of social activity by calculating the number of social activities older adults participated in. According to previous research, the study further categorized social activity into four types (65): item (1) was categorized as simple interpersonal activities (SIA), items (2) and (7) as intellectual activities (INA), items (3) and (6) as volunteer activities (VOA), and items (4) and (5) as club activities (CLA).

The economic activity of older adults was measured by their participation in work, including farm work, wage labor, business activities, and assisting with family businesses. It was assigned to 1 if engaged in work, and 0 otherwise.

In this study, information activity participation among older adults was measured by their internet usage, which was assigned to 1 if older adults used the internet and 0 otherwise.

Sleep activity status among older adults was measured by their sleep duration. Based on previous research, older adults' sleep duration was categorized into three classes (66): short sleep duration ( ≤ 5 h), moderate sleep duration (5 h ~ 9 h), and long sleep duration (≥9 h), and assigned values of 0, 1, and 2, respectively.

### 2.4 Mediators

This study included two mediating variables, analyzing the mediating effect of sleep activity between physical activity and frailty status among older adults, and the mediating effect of social activity between information activity and frailty status among older adults.

### 2.5 Covariates

The control variables selected for this study encompassed gender (0 = female, 1 = male), age (in years), hukou (0 = agricultural, 1 = non-agricultural), education level (0 = low level of education, including elementary school and below; 1 = medium level of education, including middle school graduation to vocational school; 2 = high level of education, including 2-/3-Year College/Associate degree and above) and marital status (0 = married, 1 = separated/divorced/widowed/never married).

### 2.6 Statistical analysis

Stata18.0 and SPSS26.0 were used for statistical analysis. Descriptive statistics and multiple linear regression analysis were initially conducted using SPSS 26.0. Frequency and standard deviation were used to represent categorical data, while mean and standard deviation were used for continuous data. Further mediation analysis was conducted using SPSS 26.0, employing the SPSS macro developed by Hayes (Model 4). Bias-corrected percentile Bootstrap method was utilized to estimate the 95% confidence interval of the mediating effects, with 5,000 samples extracted after controlling for gender, age, hukou, education level, and marital status. The mediating effect was considered significant if the 95% confidence interval did not include 0 (67). Lastly, the study concluded with a robustness test of the analysis results using propensity score matching (PSM) via Stata 18.0.

## 3 Results

### 3.1 Descriptive analysis

The descriptive results of the data are presented in Table 2. A total of 6,943 older participants were included in the study, with an average age of 69.1 years. Additionally, the gender proportion was balanced, with males and females accounting for 49.8% and 50.2% respectively. Moreover, the education level of the older adults was generally low, with 70.6% having the degree of primary school or below. Furthermore, a high proportion of older adults had agricultural hukou, accounting for 75.4%, and the majority of older adults were in a state of marriage, comprising approximately 77.3% of the total. The average FI of the older adults included in this study was 0.37, indicating that the overall frailty status of older adults was in the lower middle level. There were significant differences in the participation of older adults in different activities. For instance, the average MET for older adults was 3.73, with a standard deviation of 1.64, indicating that the TPA participation among older adults was moderate, but there were considerable individual differences. Moreover, older adults had a low frequency of participation in social activity, with 54.1% not participating in any social activity, 29.4% participating in one social activity, and only 16.5% participating in two or more social activities. What's more, most older adults were still involved in economic activity, accounting for approximately 61.6%. However, the participation in information activity was not ideal, with only 24.7% of older adults reporting the use of the internet in their daily lives. Lastly, the sleep duration of the older participants included in the study was relatively moderate, while 22.1% of older adults had insufficient sleep duration, and 3.85% had excessive sleep duration.

**Table 2:** Descriptive statistics of variables (*N* = 6,943).

**Variables**	**Mean/%**	**SD**	**Minimum**	**Maximum**
Frailty index	0.374	0.128	0.061	0.879
**Gender**				
Male	50.151%			
Female	49.849%			
Age	69.097	6.274	60	110
**Hukou**				
Agricultural	75.400%			
Non-Agricultural	24.600%			
**Education level**				
Low level of education	70.575%			
Medium level of education	28.201%			
High level of education	1.224%			
**Marital status**				
Separated/divorced/ widowed/never married	22.685%			
Married	77.315%			
Total physical activity	3.726	1.641	0	6.062
Social activity	0.683	0.909	0	6
**Economic activity**				
Participation	38.355%			
Non-participation	61.645%			
**Information activity**				
Participation	75.313%			
Non-participation	24.687%			
Sleep activity	0.817	0.476	0	2

### 3.2 Effects of different activity types on frailty status of older adults

Table 3 presented the effects of different types of activities on the frailty status of older adults. Model 1 included only the control variables, while Model 2 added various activity variables based on Model 1. Model 3 and 4 further analyzed the impacts of different physical activity intensities and types of social activities on frailty in older adults respectively based on the foundation. After controlling for relevant covariates, TPA exhibited a significant negative effect on the frailty status of older adults (β = −0.006, *p* < 0.01). Further exploration revealed that LPA and MPA significantly predicted lower frailty status among older adults (β = −0.005, *p* < 0.01; β = −0.003, *p* < 0.01), whereas engaging in VPA could exacerbate frailty in older adults (β = 0.002, *p* < 0.05). The statistical results verified hypotheses H1. Overall participation in social activity showed a significant negative impact on the FI of older adults (β = −0.007, *p* < 0.01). Based on this, the study further investigated the influence of different categories of social activity participation on FI. The results indicated that engaging in INA and CLA significantly contributed to lower FI among older adults (β = −0.027, *p* < 0.01; β = −0.013, *p* < 0.05), while participating in SIA and VOA did not affect frailty status. The data analysis results showed that Hypothesis 2 is partially supported. Additionally, the study findings revealed that engaging in economic activity, information activity, and sleep activity all significantly predicted lower frailty status among older adults (β = −0.017, *p* < 0.01; β = −0.040, *p* < 0.01; β = −0.044, *p* < 0.01). These findings validated hypotheses H3, H4, and H5.

**Table 3:** Effects of PA on frailty status in older adults.

**Variables**	**Frailty index of older adults**			
	**Model 1**	**Model 2**	**Model 3**	**Model 4**
Gender	−0.044^***^ (0.003)	−0.035^***^ (0.003)	−0.036^***^ (0.003)	−0.033^***^ (0.003)
Age	0.003^***^ (0.000)	0.001^***^ (0.000)	0.002^***^ (0.000)	0.001^***^ (0.000)
Hukou	−0.041^***^ (0.003)	−0.039^***^ (0.003)	−0.038^***^ (0.003)	−0.038^***^ (0.003)
Education level	−0.063^***^ (0.003)	−0.052^***^ (0.003)	−0.051^***^ (0.003)	−0.052^***^ (0.003)
Marriage	−0.022^***^ (0.003)	−0.019^***^ (0.003)	−0.019^***^ (0.003)	−0.019^***^ (0.003)
Total physical activity		−0.006^***^ (0.001)		−0.006^***^ (0.001)
Low-intensity physical activity			−0.005^***^ (0.001)	
Moderate physical activity			−0.003^***^ (0.001)	
Vigorous physical activity			0.002^**^ (0.001)	
Social activity		−0.007^***^ (0.002)	−0.007^***^ (0.002)	
Simple interpersonal activities				−0.001 (0.003)
Intellectual activities				−0.027^***^ (0.004)
Volunteer activities				0.006 (0.004)
Club activities				−0.013^**^ (0.005)
Economic activity		−0.017^***^ (0.003)	−0.022^***^ (0.003)	−0.018^***^ (0.003)
Information activity		−0.040^***^ (0.004)	−0.038^***^ (0.004)	−0.039^***^ (0.004)
Sleep activity		−0.044^***^ (0.003)	−0.044^***^ (0.003)	−0.043^***^ (0.003)
Adjustment R^2^	0.190	0.248	0.249	0.252
F-value	327.512^***^	230.009^***^	192.829^***^	181.224^***^

Standard errors in parentheses. ^**^, and ^***^ indicate significant at the 5%, and 1% levels, respectively.

### 3.3 Mediation analysis

**Figure 3:**
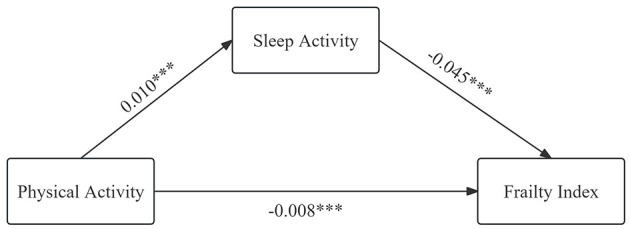
Mediating effect of sleep activity.

**Figure 4:**
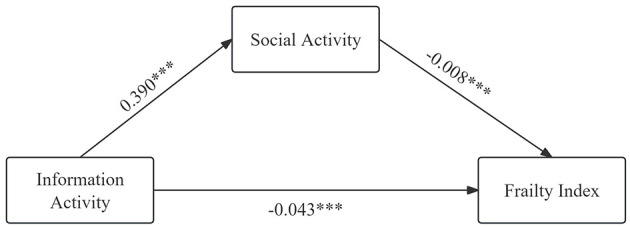
Mediating effect of social activity.

Regression analysis indicated that participation in physical, social, economic, information, and sleep activities all had varying degrees of negative effects on frailty status in older adults. However, previous studies have shown that there were also mutual influences among these activities. Therefore, further exploring the mechanisms of how different types of activities affect frailty status in older adults is of significant importance. In this study, sleep activity and social activity were selected as mediating variables to investigate the influence mechanisms of physical activity and informational activity on frailty status respectively. The results were presented in Tables 4, 5 and Figures 3, 4. Sleep activity was found to partially mediate the relationship between physical activity and the FI in older adults. The direct effect coefficient between physical activity and frailty index was −0.0083, and the mediating effect coefficient of sleep activity was −0.0004. The mediating effect accounted for 4.8% of the total effect. The mediation indicated that physical activity influenced frailty status in older adults by extending their sleep duration. Social activity partially mediated the relationship between information activity and frailty. The coefficient of direct effect between information activity and frailty was −0.0429, and the mediating effect coefficient of social activity was −0.0033. The mediating effect accounted for 7.7% of the total effect. The mediation suggested that information activity alleviated frailty status in older adults by promoting their participation in social activity. The statistical analysis results confirmed H6 and H7.

**Table 4:** Mediating effect of sleep activity.

	**Effect value**	**Boot SE**	**Boot LL CI**	**Boot UL CI**	**Relative mediation effect**
Total effect	−0.0083	0.0009	−0.0100	−0.0066	
Direct effect	−0.0079	0.0008	−0.0095	−0.0062	95.181%
Indirect effect	−0.0004	0.0002	−0.0008	−0.0001	4.819%

**Table 5:** Mediating effect of social activity.

	**Effect value**	**Boot SE**	**Boot LL CI**	**Boot UL CI**	**Relative mediation effect**
Total effect	−0.0429	0.0035	−0.0498	−0.0360	
Direct effect	−0.0396	0.0036	−0.0467	−0.0326	92.308%
Indirect effect	−0.0033	0.0006	−0.0045	−0.0021	7.692%

### 3.4 Robust test

We used two propensity score matching methods, including nearest neighbor matching and kernel matching in this study. The results were presented in Table 6. After controlling gender, age, hukou, education level as well as marital status, the effects of five different types of activities on frailty among older adults were all significant, suggesting that the baseline regression results were robust.

**Table 6:** Robust test.

	**Physical activity**	**Social activity**	**Economic activity**	**Information activity**	**Sleep activity**
Nearest neighbor matching	−0.047^***^	−0.020^***^	−0.024^***^	−0.043^***^	−0.062^***^
Kernel matching	−0.048^***^	−0.020^***^	−0.023^***^	−0.043^***^	−0.062^***^
Covariates	Control	Control	Control	Control	Control

^***^indicates significant at the 1% level.

## 4 Discussion

This study investigated the effects of physical activity, social activity, economic activity, information activity, and sleep activity on frailty in older adults and explored the potential mechanisms underlying these effects. Our findings indicated that all five activity types have significant negative effects on frailty of older adults. Specifically, physical activity can alleviate frailty by extending sleep duration, and information activity can reduce frailty by enhancing social engagement.

Initially, participation in different types of activities had a significant negative impact on frailty in older adults. First of all, the effectiveness of PA interventions in coping with frailty in older adults has been documented (25, 68). PA is defined as all movements in daily life, including occupational, commuting, and leisure activities (69). It is closely associated with maintaining and improving physiological function and physical health, and the impact of different intensity of physical activity on frailty varies. LPA such as walking, climbing stairs, and rising from the floor or chair can improve muscle strength, enhance balance and flexibility, reduce the risk of chronic diseases and musculoskeletal issues, as well as improve cognitive function and mental health in older adults (70). The improvement is closely related to the functional capacity of older adults and can significantly alleviate frailty with aging (71). MPA such as cycling and brisk walking has been shown to be relevant to lower resting blood pressure, improved lipid and lipoprotein profiles, enhanced glucose homeostasis, reduced abdominal fat deposition, as well as extended active life expectancy (72). In addition, MPA can also induce rapid expansion of the mitochondrial compartment in muscle cells, which can alleviate the frailty outcomes caused by years of sedentary habits in older adults (73, 74). However, as intensity increases, PA may not necessarily have a sustained positive impact on older adults, since VPA often increases the risk of injury and sudden death in older adults (72). VPA can also lead to fatigue, respiratory difficulties, restricted mobility, anxiety, etc. (75). As a result, moderate exercise is beneficial for the physical and mental health of older adults.

Secondly, high level of social activity participation can alleviate frailty in older adults. Social activity participation is a broad concept, including involvement in volunteering, recreational activities, interpersonal interactions, etc. (76). Activity theory suggests that social engagement can provide role support for older adults and build social relationships (77), reduce feelings of loneliness (78), as well as encourage older adults to adopt healthy behaviors, thereby improving frailty. The effects of different social activities on frailty various. As individuals age, older adults may experience the loss of friends and partners (78), which may increase their risk of social isolation and loneliness, further leading to frailty (79) and increased risk of early death (80). However, CLA can create a friendly and sociable environment. Participating in these activities can enhance social contact and interaction, enhance their sense of belonging, address their social and psychological needs (81), improve their mental health and cognitive function (82), thus further alleviating frailty. Additionally, cognition decline, as an inevitable result of aging (83), further contributes to frailty in older adults (84). While participating in INA can significantly improve older adults' memory, attention, processing speed, and enhance cognitive reserve. It is an effective intervention to improve older adults' cognitive abilities (85), which can further inhibit the progression of functional decline and reduce the likelihood of disability in older adults (86). Interestingly, this study found that SIA and VOA did not have a significant impact on frailty in older adults. This may because simple interpersonal activities, which only involve visiting or communicating with others, are unlikely to affect the physiological functions and capabilities of older adults, thus having an insignificant impact on frailty conditions. Meanwhile, the volunteering awareness among older adults in China are not enough, leading to lower participation in volunteer work, thus not significantly affecting their frailty.

Thirdly, engaging in economic activity can positively influence the health of older adults. Participating in intellectual economic activities can provide cognitive stimulation, strengthen synaptic transmission (neural plasticity), and increase cognitive reserve, thus positively affecting the maintenance or improvement of cognitive function (87). This positive impact does not diminish with age, as older employees often have experienced more cognitively challenging, allowing for more cognitive exercises. Even older adults who want to take on new tasks after retirement can enhance the function of the prefrontal cortex and further improve the cognitive function through working (88). While engaging in physical economic activities as well as physical activities generated on the way to work, such as walking and cycling, all strengthen mobility and reduce the risk of disability and physical decline in older adults, thereby alleviating frailty status (88).

Fourthly, active engagement in information activity can have beneficial effects on both the physical and mental health of older adults, thereby alleviating frailty. As age increases, the prevalence of neurocognitive disorders (such as mild cognitive impairment or dementia) continues to rise (89). These cognitive impairments often lead to disability and dependence, thus exacerbating the overall frailty condition in older adults (90). However, the Internet, as an increasingly popular information technology, can provide older adults with a new source of positive stimulation. With the deep integration of Internet-based technologies (such as smartphones, wearable devices, etc.) into daily cognitive processing, older adults using the Internet can gradually develop various forms of online cognition in their aging brains and can leverage emerging online functions to achieve cognitive abilities (91). Furthermore, using the Internet can also improve the mental health of older adults. The emergence of the Internet has brought about many new types of online activities, such as playing online games, watching videos, online shopping, social interaction, etc., all of which can help older adults achieve the purpose of entertainment and leisure, thereby improving their negative emotions (92). Additionally, as the Internet provides abundant information, some older adults can regularly search for health-related information to manage their physical and mental health, so as to improving frailty condition (93).

Fifthly, extension of sleep duration can also effectively improve the frailty condition in older adults. In recent years, sleep disorders, as a risk factor for physical frailty in older adults, have gradually attracted widespread attention (94). This is mainly because sleep disorders may over activate the HPA axis, leading to increased secretion of cortisol, thereby accelerating the degradation of muscle protein (95). Sleep disorders may also lead to dysfunction of the hypothalamic-pituitary-gonadal (HPG) axis, reducing the secretion of testosterone, thereby inhibiting the synthesis of muscle protein. These imbalanced processes of degradation and synthesis lead to the deterioration of muscle mass, which in turn leads to the core of physical weakness, namely muscle weakness (96, 97). Other possible mechanisms include chronic inflammation (IL-6, TNF-α, and CRP) (98) and imbalance in growth hormone (GH) secretion (99). According to statistics, the prevalence of sleep disorders in people over 60 years old is 42.3% (100). Sleep is a modifiable lifestyle factor, which can effectively improve the overall health of older adults, reduce frailty risks, and decrease their late-stage consequences through early adoption of effective intervention measures (101).

This study found that the above five categories of activities can indirectly alleviate their frailty status through interaction. The results indicated that sleep activity played a partial mediating role in the influence of PA on frailty in older adults. Previous research has shown that regular PA can improve the sleep quality of older adults (102). This is mainly because regular PA promotes relaxation and energy consumption in older adults, which is conducive to initiating and maintaining sleep (103, 104). However, the frequency and intensity of PA among older adults need special attention. Research has found that a three times per week exercise program predicts beneficial sleep outcomes (105, 106). Specific types of activities such as Ba Duan Jin, Tai Qi, and yoga as well as combinations of different types of exercises are known to positively affect sleep in older adults (107). Improved sleep quality further enhances older adults' cognitive abilities and reduces the risk of dementia (108), alleviating physical and mental health problems, thus positively impacting the overall frailty status of older adults (109).

Moreover, social activities play a mediating role between information activity and frailty in older adults. Putnam defined social capital as “the connections between personal social networks and the norms of reciprocity and trust that arise from them” (110). Further research has divided social capital into psychological/cognitive dimensions and network/structural dimensions (111). Network social capital includes resources obtained through social networks (111) and can be measured through social participation and informatization (112). As an “network” itself, the Internet provides older adults with effective and convenient communication channels (113). In addition to enhancing communication with family and friends, the Internet also assists older adults to establish contact with strangers, conduct in-depth online communication and offline social activities (59). Simultaneously, as a platform carrying a large amount of information, the Internet hosts online and offline organizations classified according to interests and hobbies. Older adults can accumulate social capital, alleviate loneliness and enhance their sense of belonging by joining clubs of interest and participating in activities (114). Furthermore, active social participation also helps further enhance cognitive function, curb the progression of functional decline, and reduce the likelihood of disability, thereby alleviating the overall frailty status.

Above all, we believe that in the future, older adults should seize opportunities to engage in various activities according to their own circumstances. However, it is worth noting that engaging in activities with excessively high intensity may not alleviate the frailty status. The reasons of engaging in VPA in this study were for exercise partly, while others do so out of economic necessity. For older adults exercising for health, it is advisable to reduce the intensity appropriately to enhance individual safety and physical health. For those engaged in physical labor due to economic necessity, the government should enact relevant social security policies such as providing financial assistance to relieve the financial pressure. Currently, the participation of older adults in volunteer services is not satisfactory. Consequently, the communities and related organizations should strengthen advocacy and publicity of volunteer services among older adults (115). Moreover, the proportion of older adults participating in information activities is relatively low. It is necessary to rely on family members and communities to further improve the digital literacy of older adults, promote them to integrate into the information society and empower older adults (116). For rural and remote areas having issues of the digital divide (117), government departments should increase investment in communication infrastructure construction, and mobilize enterprises actively participate in promoting the popularization and application of communication technology.

This study differs from previous single or limited researches by innovatively constructing an activity classification based on the functional capability framework for healthy aging proposed by the WHO. The classification includes five categories: physical activity, social activity, economic activity, information activity, and sleep activity. The research explores the impact of these activities on frailty and further investigates the mediating roles of sleep activity and social activity in the relationship between physical activity, information activity, and frailty. The findings not only provide a detailed overview of how various daily activities affect frailty in older adults but also offer practical pathways for preventing and mitigating frailty in daily life, aligning closely with the current promotion of healthy aging concepts. However, this study has some limitations, including the use of cross-sectional data, which may not capture the dynamic relationship between activity participation and frailty. Future research should explore these relationships longitudinally to verify the temporal effects.

## 5 Conclusion

Frailty, as a medical syndrome characterized by specific physical symptoms, significantly impacts the wellbeing of older adults and imposed considerable economic and psychological burdens on their caregivers. To explore the effective mechanisms for mitigating frailty in older adults, this study summarized previous research and utilized microdata from the CHARLS 2020, combining it with the functional capability framework for healthy aging proposed by the WHO to construct a classification of different activities. The study empirically investigated the impact of participation in these activities on frailty in older adults. The results indicated that physical, social, economic, information, and sleep activities can effectively alleviate frailty. Additionally, the study examined how physical activity and information activity improved frailty status through their effects on sleep activity and social activity respectively. These findings will enrich the existing research on frailty in older adults and provide new perspectives on improving frailty status. They will enable older adults to identify clear pathways for enhancing their health in daily life and suggest that simple, feasible activity options can significantly improve their health, which holds important practical implications for enhancing the overall wellbeing of older populations.

## Data Availability

Data are available from the China Health and Retirement Longitudinal Study (CHARLS) (http://charls.pku.edu.cn/) for researchers who meet the criteria for access to CHARLS data.
